# In vivo multimodal optical imaging of dermoscopic equivocal melanocytic skin lesions

**DOI:** 10.1038/s41598-020-80744-w

**Published:** 2021-01-14

**Authors:** V. Elagin, E. Gubarkova, O. Garanina, D. Davydova, N. Orlinskaya, L. Matveev, I. Klemenova, I. Shlivko, M. Shirmanova, E. Zagaynova

**Affiliations:** 1 Privolzhsky Research Medical University, Minin and Pozharsky Square 10/1, Nizhny Novgorod, Russia 603950; 2 Nizhny Novgorod Regional Clinical Oncology Center, Delovaya Street, 11/1, Nizhny Novgorod, Russia 603126; 3 Institute of Applied Physics of the Russian Academy of Sciences, Ulyanov Street 46, Nizhny Novgorod, Russia 603950; 4 Lobachevsky State University of Nizhni Novgorod, Prospekt Gagarina (Gagarin Avenue) 23, Nizhny Novgorod, Russia 603950

**Keywords:** Optical imaging, Fluorescence imaging, Multiphoton microscopy, Melanoma, Cancer imaging, Pathology

## Abstract

There is a wide range of equivocal melanocytic lesions that can be clinically and dermoscopically indistinguishable from early melanoma. In the present work, we assessed the possibilities of combined using of multiphoton microscopy (MPM) and optical coherence angiography (OCA) for differential diagnosis of the equivocal melanocytic lesions. Clinical and dermoscopic examinations of 60 melanocytic lesions revealed 10 benign lesions and 32 melanomas, while 18 lesions remained difficult to diagnose. Histopathological analysis of these lesions revealed 4 intradermal, 3 compound and 3 junctional nevi in the “benign” group, 7 superficial spreading, 14 lentigo maligna and 11 nodular melanomas in the “melanoma” group and 2 lentigo simplex, 4 dysplastic nevi, 6 melanomas in situ, 4 invasive lentigo melanomas and 2 invasive superficial spreading melanomas in the “equivocal” group. On the basis of MPM, a multiphoton microscopy score (MPMS) has been developed for quantitative assessment of melanoma features at the cellular level, that showed lower score for benign lesions compare with malignant ones. OCA revealed that the invasive melanoma has a higher vessel density and thicker blood vessels than melanoma in situ and benign lesions. Discriminant functions analysis of MPM and OCA data allowed to differentiate correctly between all equivocal melanocytic lesions. Therefore, we demonstrate, for the first time, that a combined use of MPM and OCA has the potential to improve early diagnosis of melanoma.

## Introduction

Although melanoma is less common than other types of skin cancers, it causes the vast majority of skin cancer deaths. Therefore, early diagnosis of melanoma and differentiating it from benign melanocytic lesions has a principal clinical importance. Dermoscopy is the first-line examination in the clinical setting, as it increases the sensitivity and specificity of diagnosis compared with a naked eye examination^1^. It has been shown that dermoscopy enables the separation of benign and malignant lesions, with the total dermoscopy score (TDS)^2^ < 4.75 and > 5.45, correspondingly, and therefore, reduces the number-needed-to-biopsy^3,4^. However, in equivocal lesions (TDS 4.75–5.45) specificity of dermoscopy is only 49%^5^. According to Ref.^6^, up to 80% of melanocytic skin lesions can be equivocal and difficult to diagnose both clinically and dermoscopically. As a result, the rate of false-positive misdiagnoses ranges from 5.5^7^ to 23%^8^ and up to 41%^9^ in some reports.

It is now well recognized that benign melanocytic nevi in certain areas of the body (acral locations, flexural skin, the scalp, the ears, face and the back) can display atypical features that might lead to a misdiagnosis of melanoma^10^. Dysplastic nevi present atypical features both clinically and histologically, and thus are important as simulants of melanoma^11,12^. Also the earliest stage of melanoma, in situ*,* being characterized by proliferation of the tumor cells within the epidermis, is less evident on dermoscopic images than invasive melanoma^13^.

In attempt to differentiate melanoma from other pigmented skin lesions, different non-invasive label-free optical techniques have been tested in recent years, including multiphoton microscopy (MPM) and optical coherence angiography (OCA).

MPM selectively visualizes the cells and the extracellular matrix on the basis of two-photon excited endogenous fluorescence from nicotinamide adenine dinucleotide (NADH), flavins, melanin, keratin, and elastin, and second-harmonic generation from collagen fibers. MPM enables in vivo real-time investigation of the skin with subcellular resolution (0.5 µm laterally and 1–2 µm in the axial direction) at the depth up to 100–200 µm^14^. It has previously been shown, that MPM is able to visualize in vivo the tumor cell nests and the effects of palisading in basal cell carcinomas of both the superficial and nodular forms^15^. It has also been possible to identify the features of squamous cell carcinoma and actinic keratosis using this technique^16^. Several studies show the possibility of using MPM for melanoma diagnosis^17–19^. On the basis of evaluation of morphological changes in melanoma relative to benign nevi and healthy skin, the sensitivity and specificity of MPM for melanoma diagnosis were calculated^17,19^. In the study by Balu et al., characteristic features of dysplastic melanocytic nevi were identified^18^. However, it seems that the only morphology analysis is not enough to differentiate between invasive and in situ stages of melanoma.

OCA, an imaging modality on the basis of optical coherence tomography (OCT), provides the possibility of real-time visualization and quantification of the blood vessels network with an imaging depth of up to 1 mm in the skin and a resolution of 3–15 µm^20^. OCA employs the analysis of the speckle variation frequency of the full complex OCT signal (amplitude and phase) of a series of pixelated OCT images taken of a same area of tissue^21,22^. In previous studies, structural features of the vessel network typical of various skin lesions have been identified^23–26^. Melanocytic nevi were characterized by regularly distributed dotted vessels. Invasive melanomas showed long linear vessels of irregular size and distribution with a branching architecture^23^. It is known that melanoma progression and invasion is accompanied by the acquisition of a denser vascular network^27^. However, in previous papers only qualitative features of vessels have been described.

We hypothesized that detection of disorders at both the cell morphology level with MPM and tissue architecture level with OCA can improve the diagnosis of melanoma in patients with equivocal skin lesions. The goal of the present work was to validate a combined use of MPM and OCA for differential diagnosis of equivocal melanocytic lesions. For this end, we firstly identified and quantitatively assessed the morphological and vessel network features of clinically diagnosed benign lesions (n = 9) and melanomas (n = 32) in patients using MPM and OCA techniques. Then the same features were assessed in 18 equivocal melanocytic lesions, which were subsequently classified using discriminant functions analysis.

## Results

### MPM and OCA features of benign lesions and invasive melanomas

**Figure 1:**
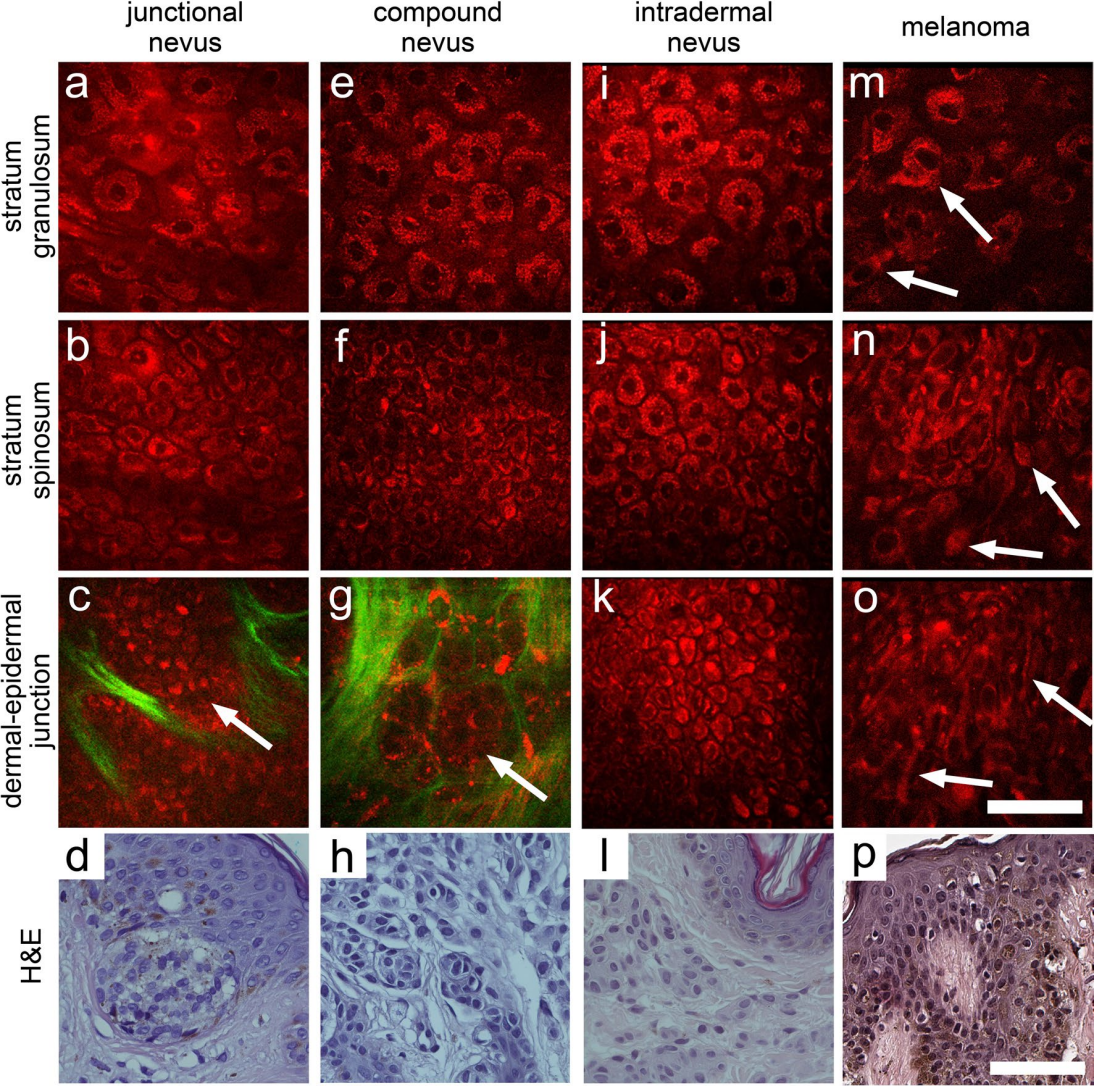
MPM of benign melanocytic lesions and melanoma. Representative MPM images of junctional (**a**–**c**), compound (**e**–**g**), intradermal (**i**–**k**) nevi and melanomas (**m**–**o**) at various depths and corresponding histopathological images (**d**,**h**,**l**,**p**). Stratum granulosum (**a**,**e**,**i**,**m**), stratum spinosum (**b**,**f**,**j**,**n**) and dermal–epidermal junction (**c**,**g**,**k**,**o**). The arrows show nevus cell nests (in **c**,**g**) and polymorphic cells (in **m**), pagetoid cells (in **n**) and dendritic structure (in **o**). Scale bar is 100 µm for all images. In MPM images, cellular autofluorescence (red) and SHG signal from collagen (green) are shown.

First, MPM images of dermoscopically diagnosed benign lesions and melanomas were analyzed to reveal morphological features of malignancy. Benign nevi were characterized by stratified epidermis, consisting of regular keratinocytes (Fig. 1a–l). The dermal–epidermal junction was represented by papillae either edged (with a rim of bright cells) or non-edged (without a rim of bright cells). The presence of nevus cell nests at dermal–epidermal junction was detected in junctional and compound nevus (Fig. 1c,g).

A focal or global loss of architecture could be detected by MPM in the epidermis of the melanomas (Fig. 1m–p). Rounded pagetoid cells, as well as dendritic structures were present in the upper layers of the epidermis. Polymorphic cells were detected in all the epidermal layers. Dermal–epidermal junction was characterized by the presence of atypical cell nests and non-edged papillae. Some areas of melanoma had nonvisible papillae at the dermal–epidermal junction.

**Figure 2:**
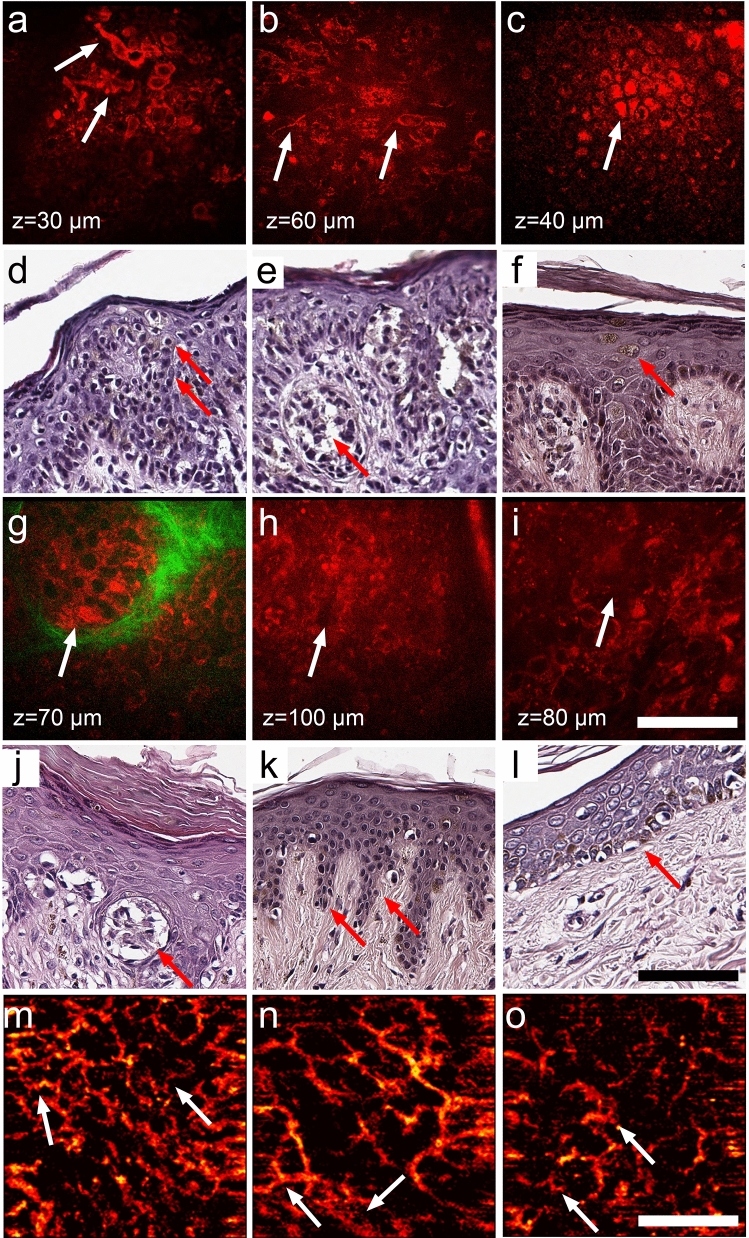
The malignant features revealed by MPM and OCA and confirmed by histopathology. The arrows show polymorphic cells (in **a**,**d**), dendritic structures (in **b**,**e**), pagetoid cells (in **c**,**f**), nests (in **g**,**j**), non-edged papilla (in **h**,**k**), nonvisible papilla (in **i**,**l**). In OCA images of vessel network the arrows show clusters of curved vessels with a diameter of ≤ 15 μm (in **m**,**o**) and clusters of irregularly distributed linear vessels with a diameter of ≥ 50 μm (in **n**). Scale bars are 100 µm for MPM and 1 mm for OCA.

On the basis of previous data^19,28^ and our observations, six features of malignancy on the MPM images were chosen for diagnostic score. They included polymorphic cells, dendritic structures, pagetoid cells, nests, non-edged papillae and nonvisible papillae (Fig. 2a–c,g–i). These features were found in histopathological specimens of the corresponding lesions (Fig. 2d–f,j–l). Two independent researchers analyzed MPM images of 21 melanomas and 10 benign lesions for the presence of selected features of malignancy. The frequency of malignant features as well as Odds Ratio (OR) were calculated for each feature. The agreement between ratings made by 2 observers on the detection of malignant features (inter-rater reliability) was estimated using Cohen’s kappa statistics with 95% confidence intervals (CI) (Table 1).

**Table 1:** Frequency of MPM malignant features in benign nevi (n = 10) and invasive melanomas (n = 21).

Features	Benign (%)	Melanoma (%)	Association coefficients, φ	OR	95% CI	*p*	Cohen’s k coefficient range
Polymorphic cells	0	19 (90.4)	0.86	163.8	7.1772; 3738.2724	0.0014	0.007–1.000
Dendritic structures	0	14 (66.6)	0.62	40	2.0811; 792.0806	0.0145	0.541–1.000
Pagetoid cells	0	15 (71.4)	0.66	50	2.5403; 987.1510	0.0101	0.195–0.981
Nests	5 (50)	8 (38.1)	0.11	0.615	0.1345; 2.8155	0.5315	0.558–1.000
Non-edged papillae	7 (70)	9 (42.8)	0.25	0.3214	0.0646; 1.6002	0.1658	0.565–1.000
Nonvisible papillae	6 (60)	13 (61.9)	0.01	1.0833	0.2319; 5.0611	0.9189	0.281–0.947

It was found that polymorphic cells, dendritic structures and pagetoid cells are typical only for melanomas, having values of association coefficient of 0.86, 0.62 and 0.66, respectively. The results of the kappa statistics analysis showed a good inter-rater reliability for these features (Table 1). While nest, non-edged papillae and nonvisible papillae are detected in both benign and malignant lesions. At that, the latter three features are more often found in benign lesions, which is likely associated with the predominance of compound nevi in the group. Therefore, these features were not included in a multiphoton microscopy score (MPMS). The MPMS calculation showed value 0 (0;0) for benign nevi and 2.14 (1.52; 2.14) for melanoma.

OCA of benign nevi and melanomas revealed that vascular network of benign nevi was present by the clusters of regular linear vessels (Fig. 2m–o). In contrast to benign nevi, densely located clusters of irregularly arborizing large caliber vessels and tortuous vessels formed a dense network in melanomas. The quantitative analysis of the OCA images confirmed these visual features. The calculated values of the vessel density and the total length of small and large caliber vessels for benign nevi and melanomas are shown in the Table 2.

**Table 2:** OCA features of benign nevi (n = 10) and invasive melanomas (n = 21).

Lesions	Total length of ≤ 15 µm diameter vessels, µm [median (25%;75%)]	Total length of ≥ 50 µm diameter vessels, µm [median (25%;75%)]	Vessel density, mm^2^ [median (25%;75%)]
Benign	1624 (1448;2512)	2320 (1416;2536)	0.055 (0.051;0.06)
Melanoma	7168 (7088;7872)	8608 (7136;11,392)	0.205 (0.174;0.21)
*p* value	≤ 0.01	≤ 0.01	≤ 0.01

The vessel density of the melanomas was significant higher than in the benign nevi (0.205 mm^2^ vs 0.055 mm^2^, *p* ≤ 0.01). The total lengths of small and large caliber vessels of benign nevi were 1624 µm and 2320 µm, correspondingly. In melanomas the total length of small and large caliber vessels increased in 4.4 and 3.7 times, respectively.

### MPM of equivocal melanocytic lesions

**Figure 3:**
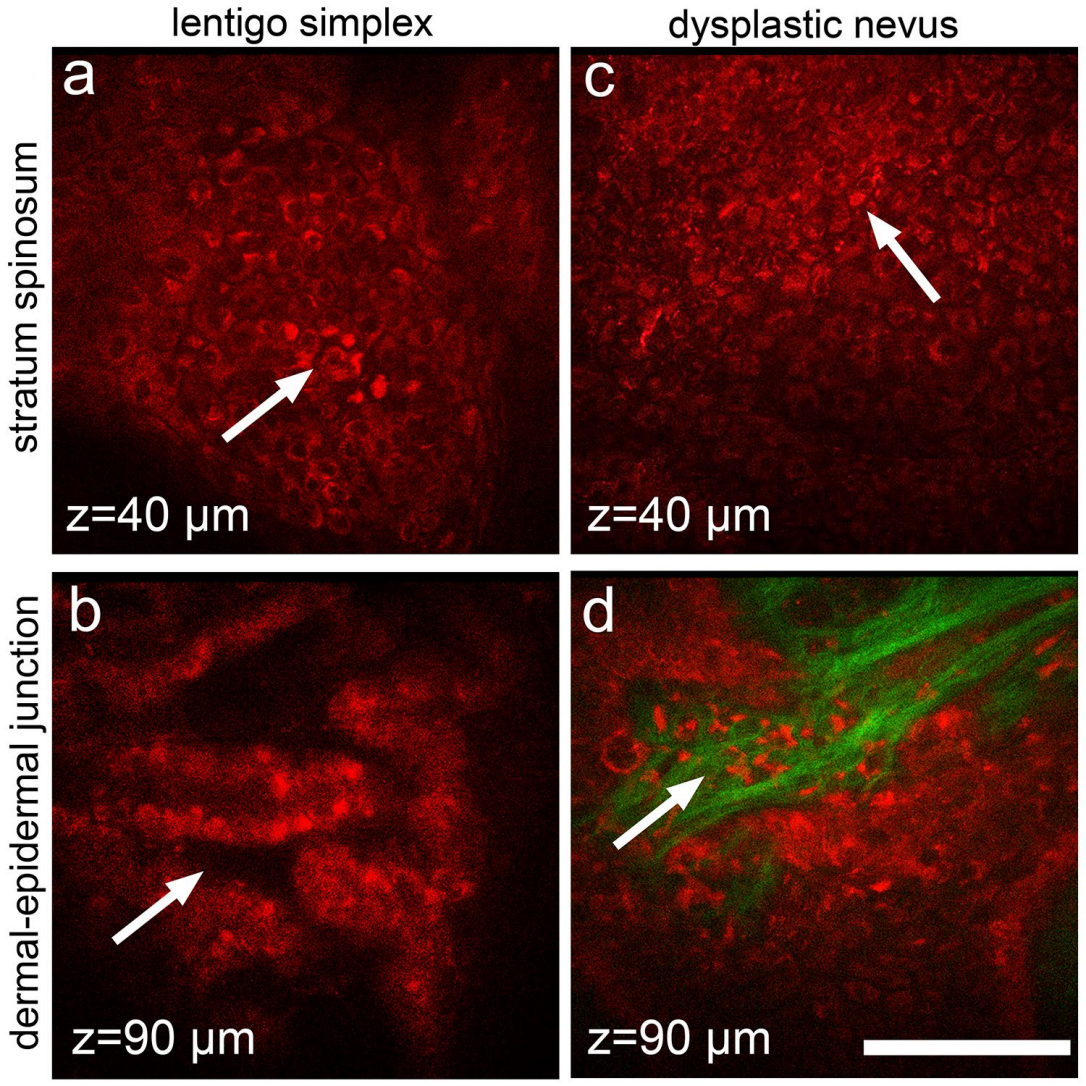
MPM features of benign lesions in dermoscopic equivocal group. In lentigo simplex (**a**,**b**), bright nucleated cells in spinous layer (**a**) and brain-like structure in dermal–epidermal junction (**b**) are shown by the arrows. In dysplastic nevi (**c**,**d**), bright pagetoid cells (**c**) in spinous layer and atypical cells in rate-ridges (**d**) are shown by the arrows. Autofluorescence from the cells (red) and SHG signal from collagen (green) are shown. Imaging depth is marked on the images. Scale bar is 100 µm, applicable to all images.

Analysis of MPM images of the equivocal lesions that were histologically benign showed mild architectural disarray of both the epidermis and the dermal–epidermal junction. However, the lesions had some features of malignancies. Specifically, pagetoid-like bright cells were detected in the stratum spinosum of lentigo simplex (Fig. 3a). These cells were also located both directly above the apex of the dermal papillae and around them. The dermal–epidermal junction was represented by elongated structures, separated by dark areas, showing a brain-like appearance (Fig. 3b). Non-edged papillae were detected in some areas of the dermal–epidermal junction. An upward spreading of bright round cells was detected in the epidermis of dysplastic nevi (Fig. 3c). Dysplastic nevi typically had small elongated nests surrounded by collagen (Fig. 3d). The dermal–epidermal junction was represented by homogeneous and thickening rete-ridges with atypical cells. As a result of the resemblance of these benign melanocytic lesions to melanoma, the MPMS values was different from 0, 0.33 (0;0.66).

**Figure 4:**
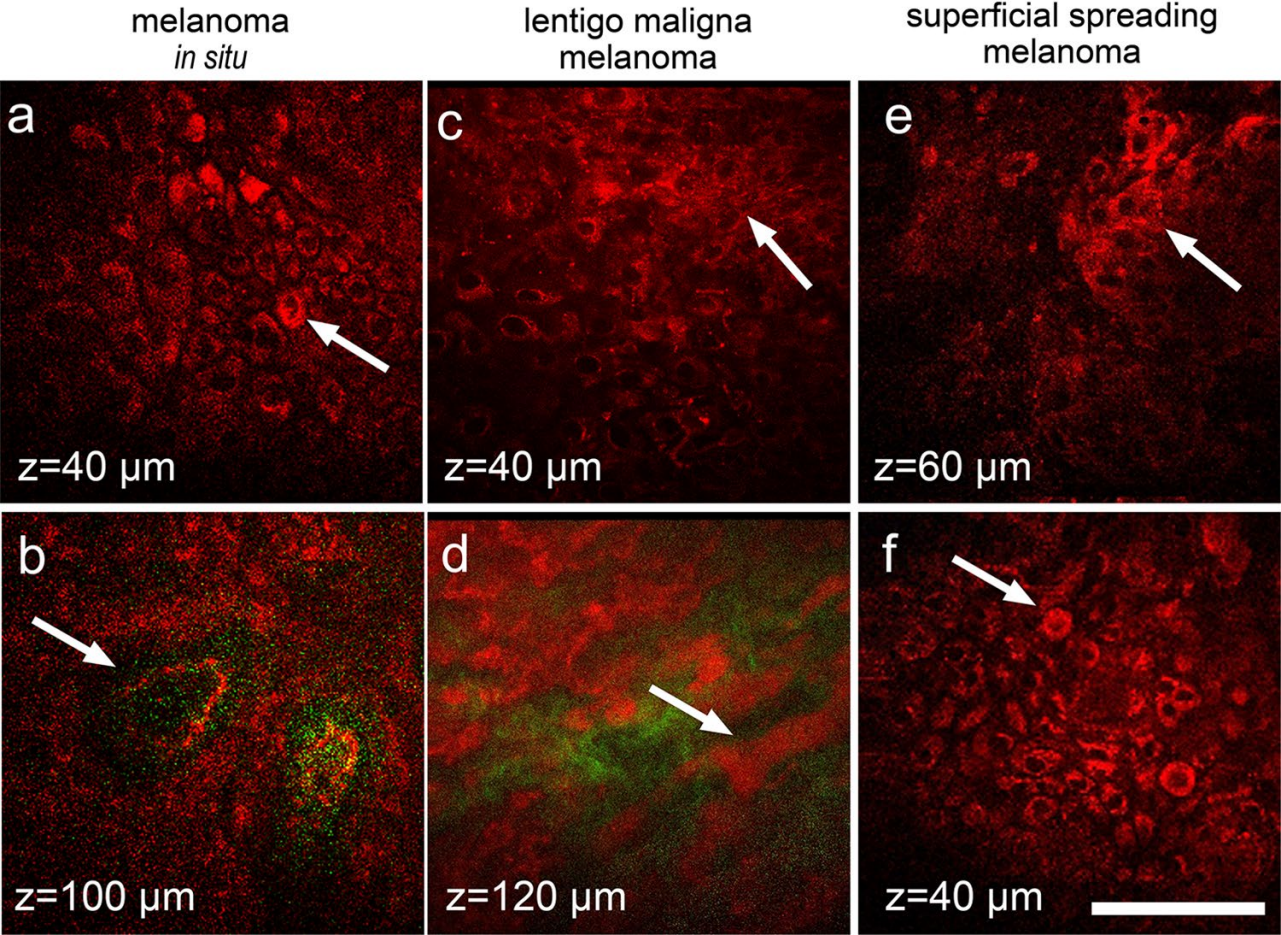
MPM features of melanoma in dermoscopic equivocal group. In melanoma in situ*,* large pagetoid cells in spinous layer (arrow, **a**) and non-edged papillae (arrow, **b**) are marked. In lentigo maligna melanoma, large pleomorphic cells in spinous layer (arrow, **c**) and melanophages below dermal–epidermal junction (arrow, **d**) are marked. In superficial spreading melanoma, nest of atypical cells (arrow, **e**) and round pagetoid cells (arrow, **f**) in the epidermis are marked. Autofluorescence from the cells (red) and SHG signal from collagen (green) are shown. Imaging depth is marked on the images. Scale bar is 100 µm, applicable to all images.

Melanomas found in the group of equivocal lesions demonstrated typical MPM features of melanomas. A focal or global loss of architecture could be detected in the epidermis of the melanoma in situ. Pagetoid cells that could also be round, large, and atypical, with evident nuclei were present (Fig. 4a). Cells with dendrite-like morphology (large and pleomorphic cells with branches) were seen in the invasive lentigo melanomas (Fig. 4c). Nests of large and bright cells with clear nuclei and homogenous cytoplasm were present in the epidermis of the superficial spreading malanomas (Fig. 4e,f). Non-edged and non-visible papillae were present at the dermal–epidermal junction (Fig. 4b). Melanophages, appearing as large bright cells with bizarre shapes, were detected in the dermis of some thin melanomas (Fig. 4d).

Invasive melanomas that were dermoscopicaly equivocal had the MPMS identical to dermoscopic melanoma, 2.14 (1.52;2.14), in contrast to melanomas in situ*,* that had slightly lower MPMS, 1.52 (1.52;1.83).

**Figure 5:**
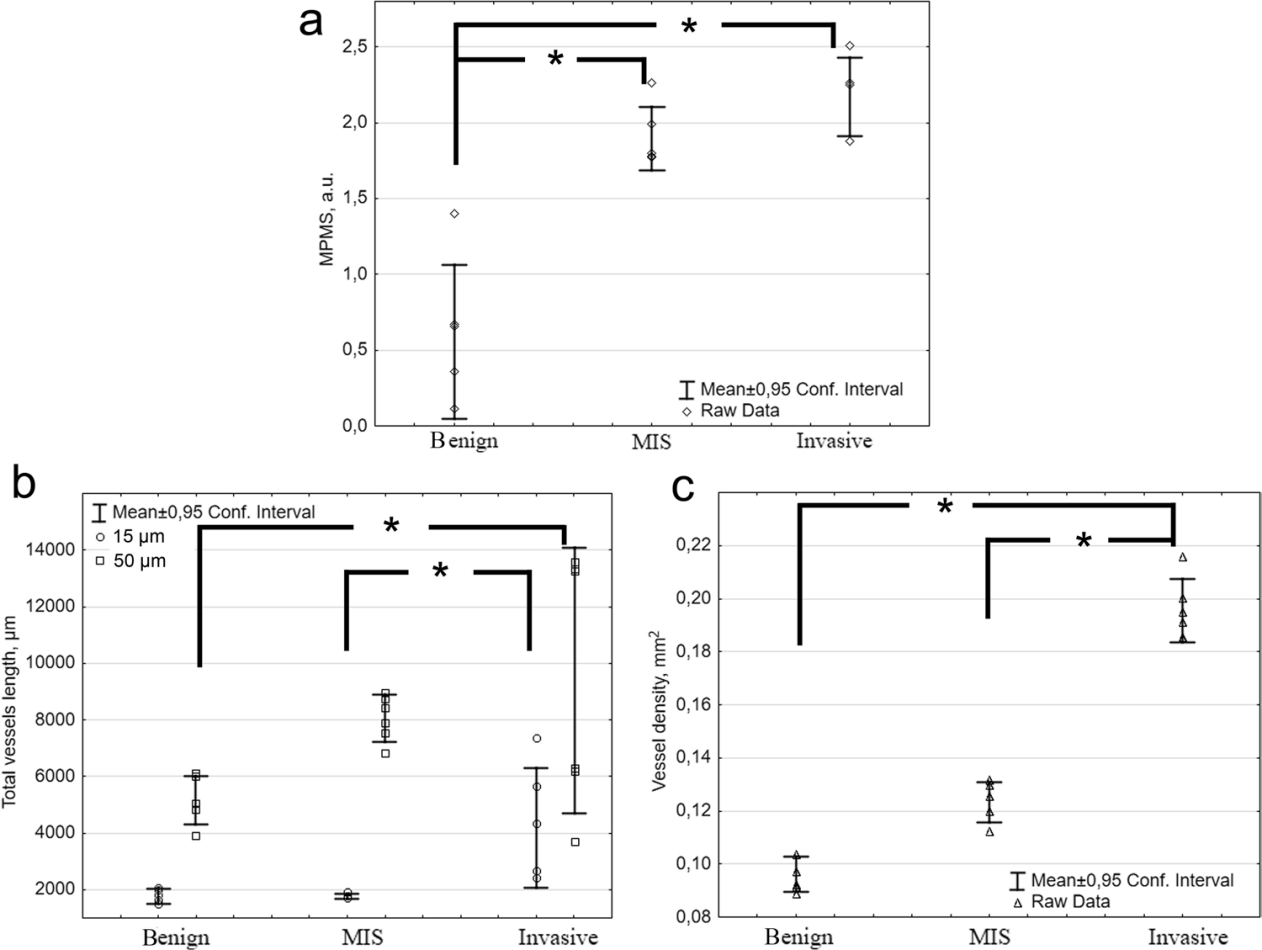
The quantitative analysis of malignant features of dermoscopic equivocal lesions revealed by MPM and OCA. The MPMS (**a**), total length of small (≤ 15 μm) and large caliber vessels (≥ 50 μm) (**b**) and vessel density (**c**) for benign melanocytic lesions, melanoma in situ (MIS) and invasive melanoma. Whiskers show the mean and 95% confidence interval. Rhombus, squares, cycles and triangles display the measurements for individual patients. *, *p* < 0.05.

Therefore, MPMS could reliably discriminate benign lesions from melanoma in situ (*p* ≤ 0.01) and invasive melanomas (*p* ≤ 0.01). However, no significant differences in the MPMS were found between in situ and invasive melanomas (Fig. 5a).

### OCA of equivocal melanocytic lesions

**Figure 6:**
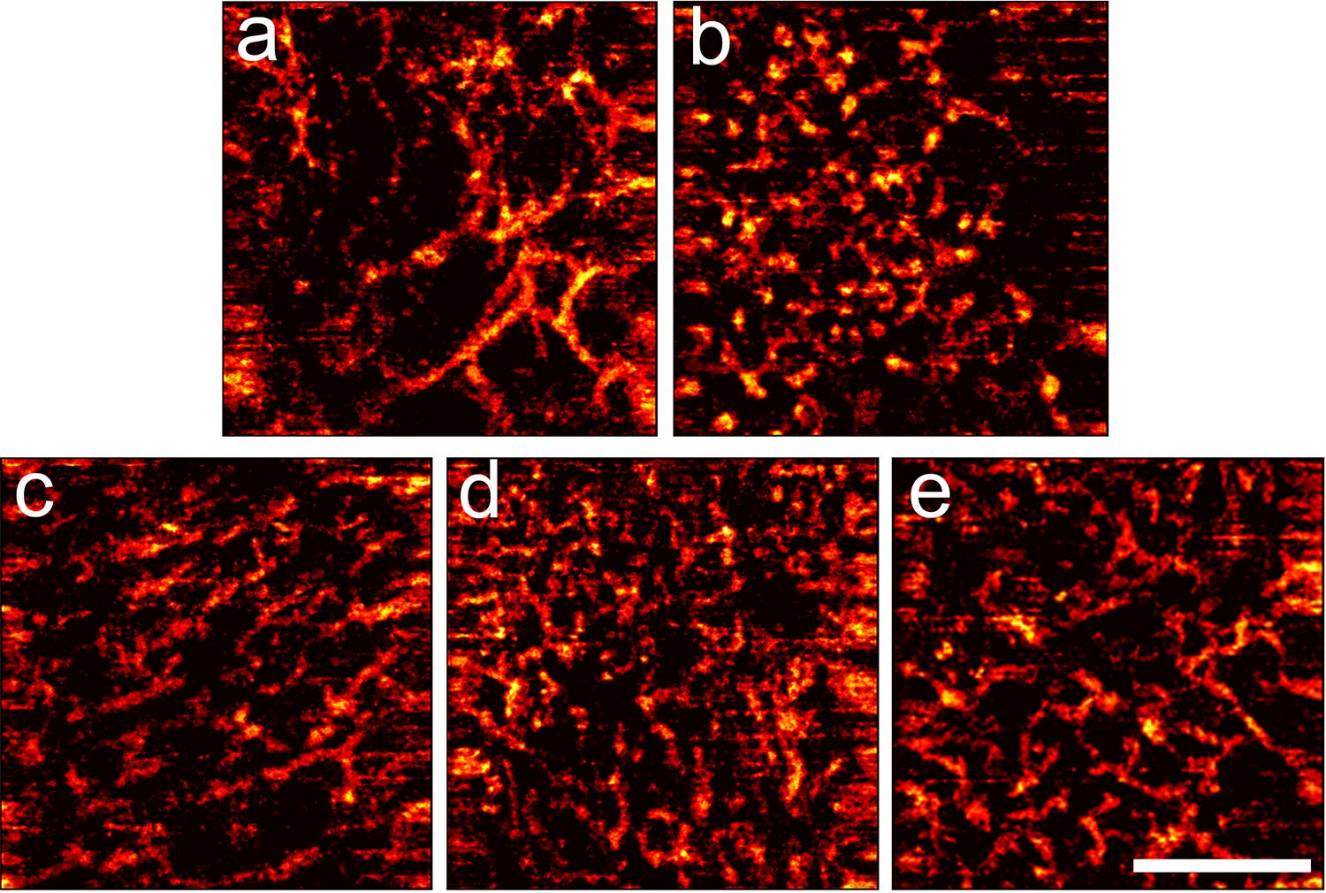
Representative OCA images of benign and malignant lesions from dermoscopic equivocal group. Vessel networks in lentigo simplex (**a**), dysplastic nevi (**b**), melanoma in situ (**c**), lentigo maligna melanoma (**d**), and superficial spreading melanoma (**e**). Scale bar is 1 mm, applicable to all images.

OCA revealed that the histologically different types of equivocal lesions had different vessel network structure. Sparely located clusters of both small and large caliber linear vessels forming a regular network could be detected in lentigo simplex (Fig. 6a). Wherein a vascular network consisting of small caliber, curved or dotted vessels was visualized in the dysplastic nevi (Fig. 6b).

A quantitative analysis of OCA images showed that the vessel density in the benign lesions of the “equivocal” group was 0.094 (0.091;0.102) mm^2^, similar to dermoscopic melanomas. This can be associated with the location of the inspected lesions. Lesions of “equivocal” group were mainly located on face while lesions of “benign” and “melanoma” groups were on trunk and extremities. It has been previously shown, that the scalp and facial skin has denser vessels network compare to other body areas^29^.

In the melanomas in situ, we observed an irregular vascular network consisting of both clusters large caliber vessels and small caliber curved vessels (Fig. 6c). OCA images of invasive lentigo melanomas demonstrated densely located clusters of irregularly arborizing large caliber vessels (Fig. 6d). A dense vascular network consisting of large caliber curved vessels was detected in the superficial spreading melanomas (Fig. 6e). The vessel density in the melanomas in situ (*p* = 0.09) and in the invasive melanomas (*p* ≤ 0.01) were higher than in the benign lesions (Fig. 5c). This mainly occurred as a result of an increase in the number of large caliber vessels (Fig. 5b). Note, the suspicions melanomas were generally more vascularized than dermoscopically diagnosed melanomas, which can be due to their location on face.

Comparison of the vessel network characteristics of melanoma in situ and invasive one showed that total length of small caliber vessels and vessels density were significantly lower in melanoma in situ (*p* = 0.011 and *p* ≤ 0.01, correspondingly), which demonstrates the prospects of OCA to differentiate between these malignancies.

### Discriminant function analysis

**Figure 7:**
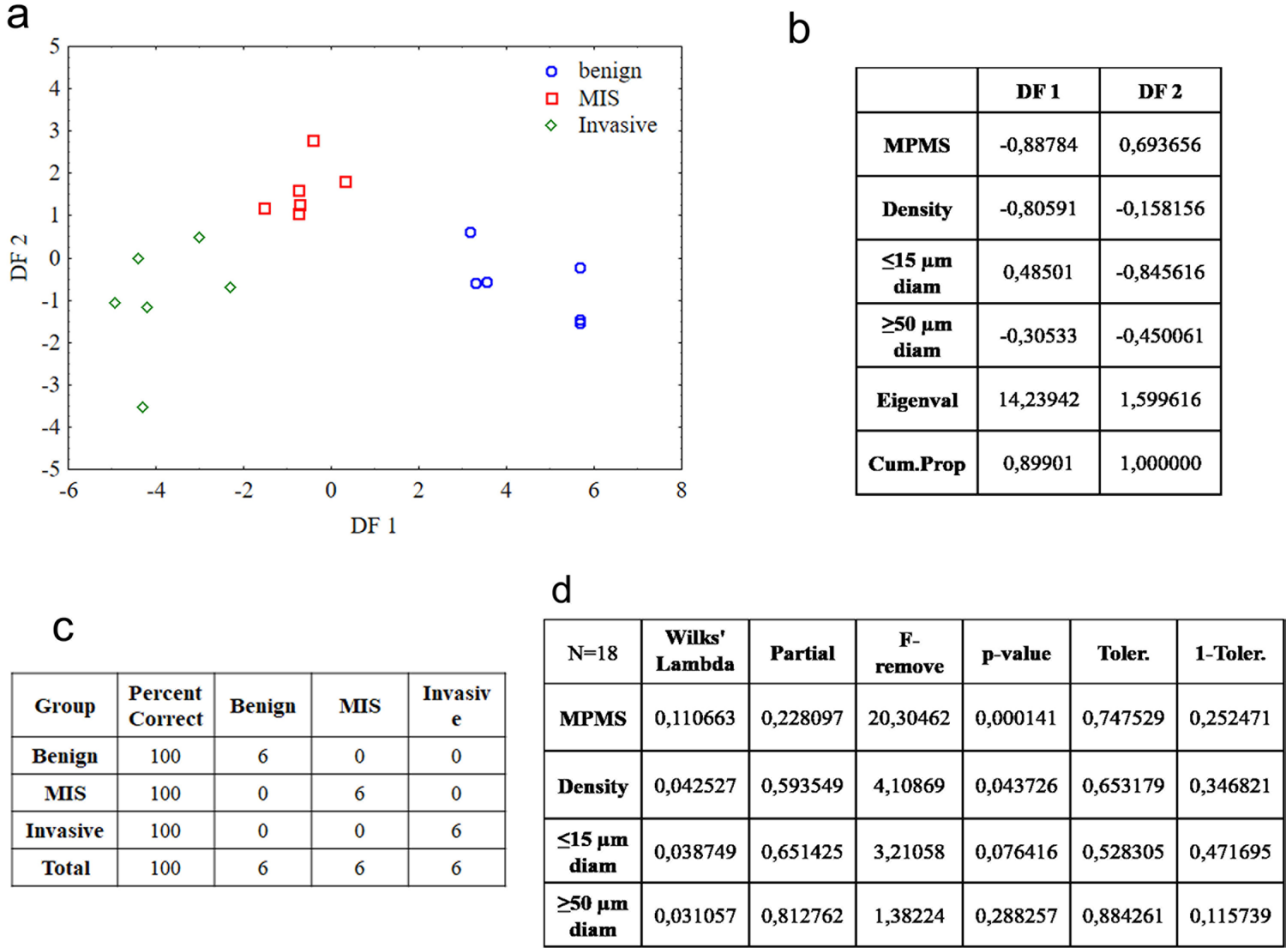
Discriminant function analysis of malignant features revealed in dermoscopic equivocal lesions. (**a**) Scatterplot represents discriminant functions (DF) distribution for benign lesions (blue cycle), melanoma in situ (red square) and invasive melanoma (green rhombus). (**b**) The standardized coefficients of discriminant functions DF 1 and DF 2. (**c**) Classification matrix. (**d**) Independent contributions to the prediction.

Discriminant function analysis was used to find out whether it is possible to classify different types of equivocal melanocytic lesions using the MPM and OCA data. Scatterplot for discriminant functions 1 (DF 1) vs. discriminant functions 2 (DF 2) allows to visualize the distribution of the cases of the equivocal lesions (Fig. 7a). It is clearly seen that DF 1 is weighted most heavily by the MPMS and OCA vessels density and the DF 2 is defined mostly by variable related to small caliber vessels length (Fig. 7b). Classification on the base of the discriminant functions allows separating benign lesions, melanomas in situ and invasive melanomas (Fig. 7c) despite that only 2 of 4 variables (MPMS and vessels density) are independent and show statistically significant *p* values < 0.05 (Fig. 7d). The small caliber vessels length has *p* value > 0.05 but as a composite of DF 2 it plays important role in the separation between melanomas in situ and invasive ones in comparison with DF 1 that has a difficulty in individually separation of melanomas in situ and invasive ones. The discriminant function analysis completely separates not only 18 dermoscopic equivocal lesions but also other studied lesions (10 benign nevi and 21 invasive melanomas).

## Discussion

Dermoscopy has a quite high level of sensitivity and specificity, however thin melanomas, especially facial localization, are difficult to differentiate from benign pigmented lesions^13^. In the present paper, we developed an approach for the in vivo diagnosis of dermoscopic equivocal melanocytic lesions, using two non-invasive optical techniques—MPM and OCA. We carried out an in vivo multimodal study of 60 melanocytic lesions in patients, of which 10 were benign, 32—malignant and 18—equivocal, according to the dermoscopic examination. Investigation of the benign and malignant lesions with MPM and OCA revealed specific features of malignancy, that were subsequently used in an examination of the equivocal melanocytic lesions.

MPT allows to examine the skin in vivo and reveal the morphological features of the tissue^14^. On the MPT images of melanomas, we identified 6 characteristic features, that were used for further analysis of equivocal lesions. The same features have been described in other studies, showing a high sensitivity and specificity^19,28^. Importantly, that the revealed MPM features for all 44 patients with melanoma were also found in histological slides. Despite the good correlation of the fluorescence and histology data, the dendritic structures could not clearly classified in the histological images. We found an association of these structures with melanoma cells (Fig. 2), however, Hashemi et al*.* has suggested that these dendritic structures are the epidermal Langerhans cells^30^. Our study shows that the identified features such as polymorphic cells, dendritic structures, and pagetoid cells have a high value of inter-expert agreement (Table 1). Nests, non-edged papillae and non-visible papillae were not only found in melanoma but also in benign melanocytic nevi. According to histopathology, these melanocytic nevi were compound nevi with elements at the dermal–epidermal junction. Moreover, the pagetoid cells and dendritic structures were also detected in benign lesion from dermoscopic equivocal group, that correlates with previous studies^18,30^. To quantify the visual MPM features, we have developed a multiphoton microscopy score (MPMS). MPMS takes into account the presence of the features on the image and their association coefficient with melanoma. The score allowed to reliably distinguish between benign lesions and melanoma, including in situ and invasive one. Note, that the MPMS values for benign lesions that were dermoscopically equivocal was higher than for dermoscopic benign lesions (MPMS 0). Melanoma in situ, present only in the group of equivocal lesions, had MPMS values lower than invasive melanomas in both equivocal and clinically diagnosed groups. Therefore, we can conclude that MPMS evaluation on the basis of MPM images made it possible to reliably distinguish benign lesions (MPMS ≤ 1.48) from melanomas (MPMS ≥ 1.52).

It should be noted that dermoscopic equivocal lesions were mainly localized on the face. The facial skin is known to be thinner than the skin of the extremities or trunk, and has the simple epidermal-dermal junction configuration^31^. In this context, such features as non-edged and non-visible papillae were excluded from the analysis. We assume that investigation of melanocytic lesions localized on the extremities or trunk would require taking into account these features.

MPT as a clinical technique has some limitations in melanoma diagnosis. Specifically, MPM provides a small size of the field of view (~ 300 µm), so that the investigation of the lesions requires series of z-stacks in the different areas, which makes the procedure rather time-consuming. In addition, the imaging depth is limited by 200 μm^28^. Therefore, melanoma thickness or tumor invasion depth can not be determined.

It is known, that angiogenesis is fundamental for tumor growth, invasion and metastasis^32^. Increased vessel density has been shown to be correlated with large histological tumor depth and decreased patient survival rate^33^. Tumor vascularity is therefore an important prognostic factor in melanomas along the tumor thickness^34^. Using OCA, both qualitative and quantitative analyses of vessels network of melanocytic lesions were performed. Specifically, we showed that the benign lesions had a sparse vessel network formed from small caliber, linear vessels. By contrast, a dense irregular vasculature consisting of large caliber, curved vessels was detected in invasive melanomas. Previously, it has been shown, using OCA, that disorganized, curved vessels and a dense vessel network is typical for melanomas, while dysplastic nevi are characterized by an increasing number of curved vessels^23^, which is consistent with our observations. Invasive melanomas were shown to contain large caliber and branched vessels, irregularly distributed. Numerous densely located dots, gradually forming irregular cloud structures were found in melanomas *in situ*^24^. However, the quantitative assessment of changes in the vessel density and thickness associated with the invasion of melanomas has not been previously performed. Our study has demonstrated, for the first time, that among dermoscopic equivocal lesions the vessel density was significant higher in invasive melanomas than in melanomas in situ and benign lesions. These features may be useful for assessments of melanomas invasion at an early stage. However, we should note that the applicability of this method and criteria of malignancy are yet to be tested to diagnosis of nodular melanomas as these forms of tumor have specific growth downwards through the skin^35^.

The values of MPMS, vessels density and vessels length were used for discriminant function analysis. The analysis was able to classify the equivocal melanocytic lesions into three distinct groups corresponding to histological diagnosis (benign lesions, melanomas in situ and invasive melanomas) on the basis of the optical features revealed by MPM and OCA.

Therefore, the developed approach, when used for differential diagnosis of melanocytic lesions, can help to reduce the number of biopsies, the workload on pathology departments and the time the patient has to wait for a diagnosis in suspicious for melanoma lesions. We continue this study to improve the proposed diagnostic algorithm and validate it with a larger number of patients.

## Conclusion

The results of this clinical study provide an initial set of in vivo MPM and OCA features of different types of dermoscopic equivocal lesions. Using MPM, we have developed a scoring system that shows potential to discriminate between benign lesions and melanomas on the basis of their autofluorescence and SHG signals. OCA has demonstrated the ability to separate melanomas in situ and invasive ones by quantitative analysis of vessels length and density. A combined use of MPM and OCA can help physicians to increase the accuracy of diagnosis of melanocytic lesions at early stage and minimize the need for biopsies.

## Materials and Methods

### Study population

Sixty melanocytic lesions of 60 patients in the Privolzhsky Research Medical University Clinic were included in our study. Exclusion criteria were the presence of erosion or ulceration of the lesion surface, nodular melanoma, as well as age of under 18 years. A routine dermoscopy examination followed by a calculation of the total dermoscopy score (TDS)^2^ was performed for all lesions by two board-certified dermatologists (G.O. and S.I.). According to dermoscopic features and TDS values, all skin lesions were divided into 3 groups: i) benign lesions (n = 10) with TDS ≤ 4.75, ii) invasive melanomas (n = 32) with TDS > 5.45 and iii) equivocal melanocytic lesions (n = 18) with TDS = 5.1 (4.9;5.4).

All lesions were examined using MPM and OCA, afterwards they underwent an excisional biopsy followed by a histopathological study (hematoxylin & eosin staining, H&E). The histopathological diagnosis was based on the consensus of two board-certified pathologists (O.N. and D.D.). Moreover, the pathologists analyzed all histological images to confirm the presence of the selected MPM features. The histopathological examination revealed 4 intradermal, 3 compound and 3 junctional nevi in the “benign” group, 7 superficial spreading melanomas and 14 lentigo maligna melanomas and 11 nodular melanomas in the “melanoma” group. Nodular melanomas were excluded from analysis due to their growing features. Benign nevi were located on extremities and shoulders. Lesions from the “melanoma” group were located on abdomen, chest, back, shoulders and extremities. The median Breslow depth of these melanomas was 0.8 (0.5;2.62) mm. In the group of equivocal lesions there were 6 benign lesions (2 lentigo simplex and 4 dysplastic nevi), 6 melanomas in situ and 6 invasive melanomas (4 lentigo melanomas and 2 superficial spreading melanomas) with the Breslow depth 0.61 (0.5;0.65) mm. Equivocal lesions were located on face, near breast, close to axillae and lumbar region.

The study was approved by the Research Ethics Board of the Privolzhsky Research Medical University. Informed consent was obtained from all participants and/or their legal guardians enrolled in the study. All methods were performed in accordance with the relevant guidelines and regulations.

### Multiphoton microscopy

In vivo examinations were performed using a commercially available multiphoton tomograph, the MPTflex (JenLab, Germany). The system consists of a femtosecond tunable titanium sapphire laser MaiTai (Spectra Physics, USA), a flexible arm with near-infrared optics and a beam-scanning module. MPTflex uses two photomultiplier tube detectors for parallel acquisition of the autofluorescence (409–660 nm) and the SHG (373–387 nm) signals. The excitation wavelength of 750 nm induced strong cellular fluorescence within the epidermis and strong SHG signal of collagen in the dermis. Ten optical sections were acquired at different tissue depths up to 100 µm with an axial step of 10 µm in the horizontal plane (z-stack). Five z-stacks containing overall 50 images were obtained for each lesion. The acquired images were 512 × 512 pixels (230 × 230 µm) in size. Image acquisition time was 6 s.

Identification of the MPM features of malignant lesions was performed in close cooperation with two dermatologists (G.O., S.I.). Next, MPM images of equivocal lesions were evaluated by 2 independent experienced biologists (E.V., G.K.) for the identification of malignancy features.

### Multiphoton microscopy score

For quantitative analysis of the malignancy features identified in MPM images, we developed a multiphoton microscopy score (MPMS). The score is based on the sum of the revealed malignancy features multiplied by their weight factors (Eq. 1). All lesions were analyzed for the presence of 3 features of malignancy: polymorphic cells (PC), dendritic structures (DS), pagetoid cells (PGC). A lesion was assigned a value of 1 for any features if such a feature was present in at least one MPM image, otherwise the lesion was scored 0 for that feature. The values of association coefficient φ were used as weighted factors of corresponding feature.

1 $$MPMS = PC \times 0.86 + DS \times 0.62 + PGC \times 0.66$$

### Optical coherence angiography

A spectral domain OCT system (IAP RAS, Russia) operating at a central wavelength of 1.3 μm and with an axial resolution of ~ 15 μm and lateral resolution of ~ 20 μm in air, with an imaging speed of 20,000 A-scans/sec was used^36,37^. The infrared laser power incident on the tissue was ~ 2 mW, and the acquisition time was 26 s. The optical probe was positioned at the skin site with gentle contact, using an articulated arm. Based on temporal speckle variations as the source of the angiographic image contrast, OCA images (2.4 × 2.4 × 1.5 mm^3^) were obtained in real-time^36^. Angiographic algorithm used image phase alignment to reduce motion artefacts, together with high-pass filtering, along the slow scanning axis^20^. Five OCA images were acquired for each lesion. Visual description of microvascular networks was performed using dermoscopic terminology^38^.

For OCA analysis and quantification, each 3D image of microvascular network was converted to a 2D *en-face* image using maximum intensity projection showing the vascular network *en-face* over the entire visualization depth of ~ 1 mm as described in Ref.^39^. We emphasize that OCA visualizes only perfused blood vessels. The resulting OCA images were binarized and skeletonized^40^.

The vessel density and the total length both of small and large caliber vessels were calculated. The small caliber vessels visible on the OCA images were ≤ 15 μm in diameter. Vessels ≥ 50 μm in diameter were classified as large caliber. The vessel density was calculated as the product of the multiplication of total number of skeletonized pixels by area of the one pixel in square micrometers^41^. Density and vessel lengths were calculated in the entire volume acquired in the dermis, over the entire visualization depth of ~ 1 mm.

### Discriminant function analysis

The data were analyzed with Multivariate Exploratory Techniques using Discriminant Function Analysis (DFA)^42^. DFA was performed to evaluate the most significant parameters for malignancy. DFA computes orthogonal discriminant functions. The maximum number of functions is equal to the number of groups minus one, or the number of variables in the analysis, whichever is smaller. In our case, two discriminant functions were estimated.

### Statistical analysis

The frequency of each feature in benign lesions, melanomas and equivocal melanocytic lesions was assessed. A contingency table 2 × 2 was used to calculate the association coefficient φ of each feature with melanoma. Odds ratios (OD), 95% confidence interval (CI) and *p* values were calculated for each feature. The agreement between ratings made by 2 observers on the diagnosis and malignant features (inter-rater reliability) was estimated using Cohen’s kappa statistics with 95% confidence intervals.

The results were expressed as median(25%;75%). To calculate the statistical significance of the differences, the ANOVA with Bonferroni post-hoc test was used. Statistical analysis was performed with Statistica 10 (StatSoft. Inc., Tusla, OK, USA). *p* values ≤ 0.05 were considered statistically significant.

## Data Availability

The datasets generated during and/or analyzed during the current study are available from the corresponding author on reasonable request.
